# Ablative Therapy for Esophageal Dysplasia and Early Malignancy: Focus on RFA

**DOI:** 10.1155/2014/642063

**Published:** 2014-07-21

**Authors:** Rebecca Tuttle, Steven J. Nurkin, Steven N. Hochwald

**Affiliations:** Roswell Park Cancer Institute Elm & Carlton Streets, Buffalo, NY 14263, USA

## Abstract

Ablative therapies have been utilized with increasing frequency for the treatment of Barrett's esophagus with and without dysplasia. Multiple modalities are available for topical ablation of the esophagus, but radiofrequency ablation (RFA) remains the most commonly used. There have been significant advances in technique since the introduction of RFA. The aim of this paper is to review the indications, techniques, outcomes, and most common complications following esophageal ablation with RFA.

## 1. Introduction

Barrett's esophagus (BE), with a reported prevalence of 1–6% in the general population, is intestinal metaplasia (IM) of the esophageal epithelium resulting from repeated mucosal injury secondary to chronic esophageal reflux [1, 2]. Histologically, this is defined as columnar replacement of the esophageal squamous epithelium with the demonstration of intestinal goblet cells [3]. BE is a premalignant condition with serial progression from metaplasia to low grade dysplasia (LGD) to high grade dysplasia (HGD) to early cancer to invasive cancer. The reported rate of Barrett's progression to malignancy is 0.5% per year [3]. Once the progression to esophageal cancer is complete, the overall 5-year survival for esophageal cancer is 38%, 20%, and 3% for localized, regional, and distant disease, respectively. This reinforces the importance of continued surveillance and aggressive treatment of Barrett's esophagus and esophageal dysplasia [4]. Although BE is widely prevalent in the U.S. population, the progression to malignancy is only found in a fraction of patients. The difficulty lies with identifying this fraction for aggressive screening.

In a recent survey of 236 gastroenterologists, the current practices for patient with Barrett's esophagus with and without dysplasia were reported. Eighty-five percent of gastroenterologists surveyed were in community practice. For Barrett's without dysplasia, 86% recommended surveillance, 12% performed ablation, and 3% offered no intervention. For Barrett's with low-grade dysplasia, 56% recommended surveillance, 26% performed ablation in all patients, and 18% performed ablation selectively. For Barrett's with high-grade dysplasia, 58% referred patients to a specialized center, 13% performed ablation in all patients, and 25% performed ablation selectively. Radiofrequency ablation (RFA) was the most common technique utilized (39%) followed by EMR (17%) [5].

Traditionally, the treatment for patients with HGD associated with Barrett's esophagus was esophagectomy given the concern for occult malignancy. However, the morbidity and mortality rates associated with open esophagectomy are 42% and 3%, respectively [6]. Even with the advent of the minimally invasive esophagectomy, the morbidity and mortality are not trivial with a reported mortality of 2% and morbidities including pneumonia (4.9%) and anastomotic leak (7.8%) [7]. The reported mortality for the endoscopic management of esophageal dysplasia and early cancer is 0.4% [8]. For this reason, the interest in endoscopic management of esophageal dysplasia and early cancer has continued to rise. In a recent review, Wu et al. investigated the difference between endotherapy and esophagectomy for the treatment of Barrett's esophagus with high-grade dysplasia and intramucosal cancer. They found no significant difference for neoplasia remission rate, overall survival at 1, 3, and 5 years, and neoplasia-related mortality risk. Patients treated with endotherapy had higher incidence of neoplasia recurrence (RR 9.50) but fewer major adverse events (RR 0.38) [9]. This reinforces the use of endoscopic ablative therapies as an alternative to the traditional surgical approach in the appropriate patient population. Here, we complete a review of the endoscopic management of esophageal dysplasia and early cancer with a focus on radiofrequency ablation and its outcomes.

## 2. Review

### 2.1. Patient Evaluation

The initial evaluation of patients with BE begins with high resolution endoscopy for thorough evaluation of disease. Patients' visible abnormalities are then characterized using the Paris classification [10, 11]. The BE segment is further characterized using the Prague-CM-classification including the length of the circumferential segment and the maximal extent of the Barrett's esophagus segment [12]. Four quadrant biopsies are taken every 1 cm for the length of the Barrett's segment [13]. Raised or nodular lesions are resected with endoscopic mucosal resection (EMR) prior to consideration for further ablative therapy. There is significant controversy regarding further preoperative imaging including CT or EUS for patients with HGD or early cancer. The likelihood of identifying metastatic or nodal disease in these patients is exceedingly low questioning the utility of additional work up and evaluation [14]. However, EUS to rule out more advanced disease is appropriate and performed in most centers prior to consideration of ablation.

Biopsy specimens should be evaluated by an experienced gastrointestinal pathologist. The Vienna classification is used for description of biopsy samples [15]. Patients are then considered candidates for further ablative therapy of flat areas of dysplasia if biopsies and EMR specimens are negative for intramucosal cancer without evidence of submucosal invasion. In addition, early cancerous lesions should be well- or moderately-differentiated without evidence of lymphovascular invasion.

### 2.2. Endoscopic Ablative Therapies

Alternative ablative techniques for esophageal dysplasia include photodynamic therapy (PDT), argon plasma coagulation (APC), and spray cryotherapy, most commonly (Table 1). In a series of 103 patients treated with photodynamic therapy followed by Nd:YAG laser during long-term follow-up, elimination rates were reported as 92.9% for LGD, 77.5% for HGD, and 44.4% for early cancer. Nd:YAG laser was used during long-term follow-up for ablation of recurrent or residual metaplasia or dysplasia. The reported overall [16] stricture rate was 30% [16]. In a subsequent partially-blinded randomized trial, Overholt et al. compared PDT to PPI therapy alone and found a 77% dysplasia response compared to 39% with PPI therapy alone [17]. Results were similar at 5 year follow-up [18]. There were no reported recurrences in the above studies with the addition of Nd:YAG laser therapy. There were 4.6% of patients presenting with subsquamous intestinal metaplasia noted on follow-up [16]. One of the major adverse effects of photodynamic therapy is the profound photosensitivity, which can last for 8 weeks [19–21]. In a direct comparison of RFA to PDT, Ertan et al. reported a complete histologic resolution of Barrett's dysplasia in 54.5% of patients undergoing PDT and 88.7% of patients undergoing RFA. There was one serious adverse event in the PDT group (esophageal perforation) with none reported in the RFA group, and the cost of PDT was five times more than the cost of RFA supporting their preferred use of RFA [22].

APC involves the passage of argon gas through an endoscope with the conduction of monopolar current through the gas. This allows for heat destruction of the topical tissues. There is no set depth of penetration for APC as compared to RFA. In a prospective, randomized trial of APC versus endoscopic surveillance, 51 patients were followed for a year after undergoing APC or surveillance alone. Initially, 25 of the 26 patients treated with APC had a 95% resolution of IM. No patients had a complete eradication of IM. At a year follow-up, 14 of 23 patients had 95% regression of IM and 9 of 23 had complete regression [23]. This study was followed with a combined analysis of two randomized trials. A total of 129 patients were included and randomized to APC versus surveillance. The initial ablation of >95% of IM was achieved in 61 of 63 patients in the APC arm. This persisted in 21 of 32 patients at long-term follow-up (>84 months). The progression to HGD was 1 in the APC group compared to 3 in the surveillance group. Similarly, the progression to LGD was 1 in the APC group compared to 6 in the surveillance group [24]. Several studies have been completed comparing APC to PDT for the eradication of both intestinal metaplasia and dysplasia. Results from all have demonstrated similar responses for both therapies with a slightly improved response with PDT but at a higher overall cost [25–27].

Cryotherapy has also been used for the ablation of esophageal dysplasia and early cancer. It has been proven to be a safe and well-tolerated form of therapy [34, 39]. Gosain et al. reported on 32 patients with HGD in the setting of BE who underwent spray cryotherapy. 100% of patients had complete eradication of HGD at 2 years, and 84% of patients had complete eradication of IM in the same time period. Recurrence of HGD was documented in 6 patients with all but 1 patient achieving repeat complete eradication with subsequent treatments. The reported stricture rate was 9% [35]. In a larger study, Shaheen et al. evaluated 98 patients with HGD. After 10.5 months of follow-up, reported responses were 97% for eradication of HGD, 87% for eradication of dysplasia, and 57% for eradication of metaplasia. Buried glands were found in 3% of patients [36]. In a study examining the recurrence of dysplasia following spray cryotherapy, 30% of patients developed recurrent disease in a median of 6.5 months with just under 10% going on to develop a second recurrence. Ninety-two percent of these patients were able to go on to achieve complete response with repeat therapy [40].

## 3. RFA

### 3.1. Indications for RFA

RFA's primary indication was dysplastic BE, but given the believed natural history of nondysplastic BE progressing to dysplastic BE, there are some who advocate the use of RFA for all patients with BE. Hur et al. examined the effectiveness and cost effectiveness of surveillance endoscopy with surgery when cancer was detected, surveillance with RFA when HGD was detected, and initial RFA when Barrett's was detected followed by endoscopic surveillance. For HGD, they found RFA to be more effective and cost effective than surveillance followed by surgery. For nondysplastic Barrett's, they found surveillance until HGD to be more effective and cost effective than initial RFA. For LGD, they stated that for stable disease, RFA may be effective as an initial treatment, but the data was not conclusive [41].

Pohl et al. examined the cost effectiveness in the United Kingdom of endoscopic therapy verses esophagectomy for early esophageal cancer. The reported costs of endoscopic therapy were $17,000.00 with 4.88 quality-adjusted life years. The costs for esophagectomy were $28,000.00 with 4.59 quality-adjusted life years. Varying the recurrence rates following endoscopic therapy did not affect the outcomes. The risk of lymph node metastasis with esophagectomy needed to exceed 25% for esophagectomy to be the favored treatment option [42]. Similarly, cost effectiveness models comparing RFA to esophagectomy for patients with high grade dysplasia have demonstrated improved cost effectiveness for RFA [43].

### 3.2. Method for Radiofrequency Ablation

**Figure 1:**
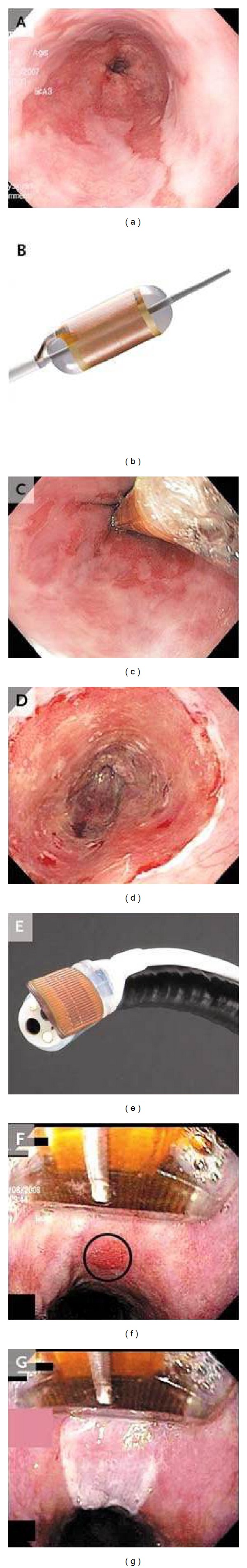
Radiofrequency ablation in the Treatment of Barrett's Esophagus [29]. Figure copied with permission from Shaheen et al. (a) Endoscopic photograph of Barrett's esophagus. (b) Circumferential radiofrequency ablation balloon. (c) Endoscopic photograph of deflated RFA balloon within the esophagus prior to ablation. (d) Endoscopic photograph of the esophagus with immediate post-RFA treatment effect. (e) Focal radiofrequency ablation device. (f) Endoscopic photograph of residual Barrett's esophagus (circled) 2 months status after ablation. (g) Endoscopic photograph of residual Barrett's esophagus status after repeat ablation [29].

The most commonly used method for esophageal RFA is circumferential balloon ablation using the Barrx system, formerly the HALO system (Covidien, Dublin, Ireland). Initial circumferential RFA is completed with the Barrx^360^-balloon. The RF energy is directed uniformly to a depth of 0.5 mm. The balloons are available in varying sizes to allow for complete tissue contact. Following circumferential RFA, or in the absence of circumferential BE, the Barrx^90^-balloon or the Barrx^60^-balloon can be utilized for site-directed radiofrequency ablation (Figure 1). Van Vilsteren et al. reported alternative methods for circumferential RFA. The standard procedure involves circumferential RFA, removal of the device for a cleaning phase, and then a second circumferential RFA. Van Vilsteren and colleagues compared this standard approach to cleaning the device without removal and to two circumferential ablations without cleaning. Ultimately, they found that the procedure could be modified to forego or simplify the cleaning phase without effect on outcomes [44]. These procedures are generally performed on an outpatient basis and require conscious sedation only. Post procedure, patients are recommended to continue a liquid diet for 24 hours. Patients may experience chest pain and dysphagia following the procedure and are generally provided with liquid pain medications prior to discharge. RFA may be repeated every 2 to 3 months until complete resolution of dysplasia and intestinal metaplasia. Following the final RFA treatment, patients should be reexamined in 2 months with biopsies taken to confirm complete resolution. Ongoing surveillance schedules following complete resolution are variable, but most commonly patients are seen every 6 months for a total of one year and yearly thereafter [45].

### 3.3. Addition of EMR

The Barrx system requires flat epithelium to maintain tissue contact and complete uniform ablation [28]. For this reason, RFA alone cannot be used to treat nodular BE or early esophageal cancer. The addition of EMR prior to ablation broadens the application of RFA. Pouw et al. completed a multicenter, prospective cohort study of 24 patients with Barrett's esophagus and HGD or early cancer. Visible lesions were resected with EMR followed by RFA of remaining intestinal metaplasia. EMR was repeated for persistent areas of intestinal metaplasia following RFA. Complete eradication of neoplasia was reported in 95% initially and 100% following repeat “escape” EMR. Complete eradication of metaplasia was reported in 88% initially and 96% following repeat EMR. After a median follow-up of 22 months, no neoplasia recurred [Pouw 2010]. Kim et al. completed a retrospective analysis of EMR prior to RFA in nodular lesions. They identified 65 patients treated with EMR prior to RFA and found no difference in efficacy or safety outcomes with EMR prior to RFA in nodular Barrett's compared to RFA alone in nonnodular Barrett's [46]. Further advancement of this technique has allowed RFA use in the setting of early intramucosal adenocarcinoma. EMR is essential in these patients to adequately characterize the lesion and ensure the absence of advanced esophageal adenocarcinoma. At the completion of EMR, all mucosal surfaces should be flat and residual areas of dysplasia should be biopsied to ensure the absence of invasive cancer. RFA may then be completed 6 to 8 weeks following EMR.

### 3.4. Outcomes of RFA Therapy

The AIM Dysplasia Trial was a multicenter, sham-controlled trial of 127 patients with dysplastic BE. Patients were assigned to either RFA or sham procedure. Outcomes analyzed included complete eradication of dysplasia and IM (Table 2). In the intention to treat analysis, 90.5% of patients with low grade dysplasia had complete eradication compared to 22.7% in the sham control group. Similarly, 81% of patients with HGD had complete eradication of dysplasia compared to 19% of patients in the sham control group. 77.4% of patients had complete eradication of IM compared to 2.3% in the control group. The RFA group also had less disease progression and fewer cancers (1.2% versus 9.3%) [29].

The AIM II Trial examined the use of RFA in nondysplastic BE. This study reported on 70 patients with short-segment BE and followed them for 2.5 years following therapy. Circumferential ablation was completed initially and repeated 4 months later if there was residual disease present. Patients were all maintained on PPI therapy after procedure. Completed eradication of IM was present in 70% of patients at 12 months and 98% of patients at 30 months with repeated treatment at 12 months if IM was detected. No serious adverse events were reported [37]. Follow-up after 5 years demonstrated complete eradication of BE in 92% of available patients with those who experienced recurrence or residual disease obtaining a complete response with repeat treatment. Again, no adverse events were reported [38].

Ganz et al. conducted a multicenter U.S. registry at 16 academic and community centers over a 3-year period. Patients with HGD and IM were included. A total of 142 patients underwent circumferential RFA, with a median of 1 session. No serious adverse events were encountered, 1 patient had an asymptomatic stricture, and there was no evidence of buried glands. Complete response of HGD was reported for 90.2% of patients, complete response of dysplasia was reported for 80.4% of patients, and complete response on IM was reported for 54.3% of patients [28].

The above studies were primarily conducted at tertiary referral centers. Lyday et al. conducted a multicenter registry in community-based gastroenterology practices. They performed step-wise RFA with subsequent follow-up and biopsies. There were a total of 429 patients treated with and without dysplasia. No serious adverse events were reported. Surveillance immediately following treatment demonstrated complete eradication of dysplasia in 89% and complete eradication of IM in 72%. Those patients followed for at least one year following therapy had complete eradication of dysplasia in 100% and complete eradication of IM in 77% with a median follow-up of 20 months [30] (Table 3).

### 3.5. RFA in the Treatment of Long and Ultra-Long Barrett's Esophagus

There has been questions on the use of RFA with or without EMR for the treatment of ultra-long segment BE (>8 cm). Dulai et al. reported similar outcomes for patients with both long segment Barrett's (3 to 8 cm) and ultra-long segment Barrett's, with eradication rates for dysplasia (>88%) and IM (>77%) for both. Patients with ultra-long segment BE did require more RFA sessions and had decreased durability of eradication, 65% versus 82%. On multivariate regression analysis, increasing Barrett's length was associated with a reduced likelihood of eradicating metaplasia but not dysplasia [47]. Herrero et al. presented similarly successful results for lesions greater than 10 cm. They had complete response of neoplasia in 83% and complete response of IM in 79% without any serious complications. Minor complications were reported in 15% [48].

### 3.6. Impact of Fundoplication on RFA

There were initial concerns over the impact that prior fundoplication would have on the ability to complete RFA safely and the expected outcomes. Shaheen et al. queried the U.S. RFA Registry for patients with and without prior fundoplication. They identified 5,537 total patients undergoing RFA and 301 patients who had undergone prior fundoplication. In their review, rates of complete eradication of IM and dysplasia were similar in both groups. Complications were also not statistically different [49]. There have been reports of improved response durability with fundoplication added to ablative therapy [50].

### 3.7. Complications of RFA

The most common complication associated with esophageal RFA is stricture formation with a reported incidence of 5–9% [29, 30, 51–53]. Hemorrhage has also been reported, although less commonly [29]. These are both generally managed endoscopically.

At the outset of RFA, there were initial concerns for postprocedure esophageal dysmotility and function. Beaumont at al. reported on the effects of RFA on inner esophageal diameter, compliance and motility. They followed 12 patients with intramucosal adenocarcinoma or HGD who underwent EMR of visible lesions followed by a maximum of 5 RFA sessions. All patients in their study had complete eradication of dysplasia and IM. Lower esophageal sphincter pressure and length and esophageal contraction amplitude were not affected by RFA. Esophageal compliance was noted to be decreased in patients with BE when compared to healthy volunteers, but not affected by RFA [54]. Similar reports have demonstrated no significant changes in esophageal motility or 24-h pH-impedance measurements before and after RFA [55].

### 3.8. Esophageal Resistance to Ablation

The reasons for resistance to RFA are not entirely clear. Poor response is defined as less than 50% reduction in Barrett's after 3 months from the initiation of therapy [56]. Gupta et al. reported patients' age and length of the Barrett's segment to be associated with longer time to complete remission of Barrett's changes [57]. Similar studies have also identified length of Barrett's segment, size of hiatal hernia, and incomplete healing between RFA treatments to be associated with increased failed therapy [53, 58]. Zeki et al. suggested that clonal selection via the RFA allows for the subsequent overgrowth of resistant clonal populations and the clinical picture of recurrent or resistant BE [59]. Looking further at patient characteristics predictive of poor response to RFA, van Vilsteren et al. identified 278 patients from 14 centers who underwent circumferential balloon-based RFA. Characteristics they identified as predictive of poor response were active reflux esophagitis, endoscopic resection scar regeneration with Barrett's epithelium, esophageal narrowing pre-RFA, and years of neoplasia pre-RFA [56]. This helps to highlight those patients who would be at increased risk for failure of therapy.

### 3.9. Recurrence following Ablation

Recurrence of BE following complete eradication is reported as 5% per year [60]. Korst et al. described 3 distinct patterns of recurrence following RFA of BE: endoscopically invisible intestinal metaplasia underneath neosquamous epithelium, visible recurrence in the tubular esophagus, and intestinal metaplasia of the gastroesophageal junction [61]. There has been significant discussion of the concept of “buried glands,” or intestinal metaplasia beneath neosquamous epithelium. Concerns include inadequate surveillance, sufficient depth of biopsy, and increased malignant potential.

There has been investigation into esophageal acid exposure and its impact on RFA effectiveness. A retrospective analysis of 45 patients' status after RFA was completed with esophageal pH monitoring. Twenty-nine percent of patients exhibited severe esophageal acid exposure despite adequate therapy. Additionally, they found that those patients with normal esophageal acid exposure had greater reduction in BE surface area and increased rates of complete eradication [62]. Similarly, Krishnan et al. completed a prospective review of 37 patients and found that predominantly weak acidic reflux despite twice daily therapy was associated with an increased incidence of persistent IM after ablation in patients with BE [58]. O'Connell and Velanovich reported on the durability of response to RFA with and without fundoplication. While the study included only 47 patients with long-term follow-up, they found that patients who had undergone fundoplication were more likely to have a durable response than those treated with proton pump inhibitor therapy [50]. Based on these data, it is likely that persistent acid reflux predisposes to recurrent BE after RFA.

### 3.10. Mucosal Resistance following RFA

Mucosal resistance is a concept introduced in those patients undergoing esophageal RFA. There was a question regarding the ability to adequately survey the postablative esophagus secondary to scarring and subsequent fibrosis. This is in concert with the concept of “buried glands” or subsquamous IM. Following RFA, the development of neosquamous epithelium can allow for the collection of metaplastic nests of tissue beneath the epithelial lining of the esophagus. First, Overholt et al. analyzed the depths of esophageal biopsies before and after RFA to determine the percentage that contained lamina propria and, therefore, an adequately representative sample. Approximately 90% of biopsies in both ablated patients and ablation-naïve patients were adequate to detect all evident intestinal metaplasia [63]. Similarly, follow-up of the AIM Dysplasia Trial demonstrated adequate surveillance biopsies in 80% of patients with the finding that biopsies on squamous surfaces were more likely to be inadequate than biopsies on columnar surfaces [64]. Yuan et al. reported a 15% incidence of buried IM following radiofrequency ablative therapy for BE. All of those patients were detected with standard biopsy and all IM underwent complete response following new or repeat RFA [65]. The 5-year follow-up of the AIM II Trial reported 85% of biopsies to contain lamina propria with no patients demonstrating buried glands [38].

### 3.11. Quality of Life following RFA

Shaheen et al. were the first to examine quality of life following RFA of the esophagus. This was analyzed from the AIM Trial in which patients were treated with RFA or sham therapy. In this population of patients, they found that, prior to any treatment, patients were worried about esophageal cancer and esophagectomy. Similarly, there were increased reports of depression, worry, and stress. Twelve months following therapy, those patients treated with RFA had significantly less worry and depression owing to the perceived benefit of RFA on the treatment of esophageal disease and the decreased risk of esophageal cancer [66].

## 4. Conclusion

Endoscopic intervention continues to be a safe and preferred alternative to radical surgery for esophageal dysplasia and intramucosal carcinoma. The most common practice pattern for the use of ablative therapies, particularly RFA, is following endoscopic mucosal resection for the assessment of any invasive malignancy. RFA has reported results superior to APC and PDT. It is generally well-tolerated with few serious, adverse events reported. When compared to PDT, RFA is more cost effective [22] and foregoes the subsequent photo sensitivity. While cryotherapy has acceptable initial results, there has been a suggestion of a higher recurrence rate (up to 30%) associated with the procedure [40]. Despite the method utilized for complete eradication of metaplasia and dysplasia, patients remain at risk for recurrence of disease. For this reason, continued surveillance is warranted.

## Figures and Tables

**Table 1:** Ablative techniques.

Esophageal ablative technique	Method	Frequency of treatment	Complete eradication of intestinal metaplasia	Complete eradication of low grade dysplasia	Complete eradication of high grade dysplasia	Common complications (%)
RFA^1^	Endoscopic balloon creates high frequency current which causes topical destruction of tissues	q2-3 months until resolution	54–97%	80–100%	81–90%	Stricture (2–6%), hemorrhage

PDT^2^	Photosensitizer given prior to procedure allows the application of a specific wavelength of light to cause creation of oxygen free radicals and topical destruction of tissues	1-2 treatments, often followed by Nd:Yag laser therapy	52%	93%	77%	Photosensitivity, Stricture (30%)

APC^3^	Argon gas is passed through endoscope, monopolar current conducts through gas, and resulting heat causes topical destruction of tissues	q8 week treatments	0–55%	—	76%	Stricture (15%)

Cryotherapy^4^	Topical application of coolants causes destruction of tissues	3–5 treatments	57–84%	87%	97%	Stricture (9%)

Description of common ablative techniques ^1^[28–31] ^2^[16, 17] ^3^[23, 26, 32, 33] ^4^[34–36].

**Table 2:** Primary and secondary outcomes at follow-up∗.

Outcome and analysis	Radiofrequency ablation	Sham procedure	Relative risk (95% CI)	*P* value	Number needed to treat^†^
	no./total no. (%)				
*Primary outcome *					
Complete eradication of intestinal metaplasia (all patients)					
Intention-to-treat	65/84 (77)	1/43 (2)	33.3 (4.8–231.7)	<0.001	1.3
Per-protocol	65/78 (83)	1/39 (3)	32.5 (4.6–225.5)	<0.001	1.2
Complete eradication of dysplasia (low-grade dysplasia)					
Intention-to-treat	38/42 (90)	5/22 (23)	4.0 (1.8–10.7)	<0.001	1.5
Per-protocol	38/40 (95)	5/19 (26)	3.6 (1.7–7.7)	<0.001	1.5
Complete eradication of dysplasia (high-grade dysplasia)					
Intention-to-treat	34/42 (81)	4/21 (19)	4.2 (1.7–10.4)	<0.001	1.6
Per-protocol	34/38 (90)	4/20 (20)	4.5 (1.8–10.8)	<0.001	1.4
*Secondary outcomes *					
Complete eradication of intestinal metaplasia (high-grade dysplasia)					
Intention-to-treat	31/42 (74)	0/21	ND	<0.001	1.4
Per-protocol	31/38 (82)	0/20	ND	<0.001	1.2
Complete eradication of intestinal metaplasia (low-grade dysplasia)					
Intention-to-treat	34/42 (81)	1/22 (4)	17.8 (2.6–121.5)	<0.001	1.3
Per-protocol	34/40 (85)	1/19 (5)	16.1 (2.4–109.3)	<0.001	1.3
Complete eradication of dysplasia (all patients)					
Intention-to-treat	72/84 (86)	9/43 (21)	4.1 (2.3–7.4)	<0.001	1.5
Per-protocol	72/78 (92)	9/39 (23)	4.0 (2.2–7.1)	<0.001	1.4
Progression of dysplasia					
Any	3/84 (4)	7/43 (16)	02 (0.1–0.8)	0.03	7.9
Low-grade to high-grade	2/42 (5)	3/22 (14)	0.3 (0.1–1.9)	0.33	11.3
Low-grade to cancer	0/42	0/22	ND	ND	NA
High-grade to cancer	1/42 (2)	4/21 (19)	0.1 (0.01–1.0)	0.04	6.0
High-grade or low-grade to cancer	1/84 (1)	4/43 (9)	0.1 (0.01–1.1)	0.045	12.3
Biopsy specimen free of intestinal metaplasia at 12 mo					
All patients	2670/2724 (98)	673/1164 (58)	1.7 (l.6–1.8)	<0.001	NA
Low-grade-dysplasia subgroup	1228/1260 (98)	313/550 (57)	1.7 (1.6–1.8)	<0.001	NA
High-grade-dysplasia subgroup	1442/1464 (98)	360/614 (59)	1.7 (1.6–1.8)	<0.001	NA
*Secondary outcomes *					
Chest-pain score on day 1^‡^					
All patients				<0.001	NA
No. of patients	81	40			
Median	23	0			
Interquartile range	0–51	0-0			
Low-grade dysplasia				<0.001	NA
Number of patients	40	20			
Median	26	0			
Interquartile range	4–48	0-0			
High-grade dysplasia				<0.001	NA
Number of patients	41	20			
Median	22	0			
Interquartile range	0–57	0-0			

*NA denotes not applicable, and ND not done.

^†^The number needed to treat refers to the number of patients who would need to be treated with radiofrequency ablation to prevent one out-come failure (the inverse of the absolute risk reduction).

^‡^Chest pain was measured on a visual-analogue scale of 0 to 100, with higher scores indicating a greater severity of pain.

Figure taken with permission from Shaheen et al. [29].

**Table 3:** Esophageal RFA results summary.

Series	Mean/*median* length of BE (cm)	Mean/*median* number of treatments required	Follow-up interval (months)	Complete eradication of intestinal metaplasia (%)	Complete eradication of low-grade dysplasia (%)	Complete eradication of high-grade dysplasia (%)	Rates of progression	Complication rates (%)
Ganz et al., 2008 [28]	*6 *	*1 *	12	54.3	80.4	90.2	—	0.7
Fleischer et al., 2008 [37]	3.2	1.5	30	97	—	—	—	0
Shaheen et al., 2009 [29]	5.3∗	Up to 4	12	77.4	90.5	81.0	4	6.0
Fleischer et al., 2010 [38]	3.2	3.4	60	92	—	—	—	0
Lyday et al., 2010 [30]	*3 *	1.8–2.1	20	77	100	—	—	1.1

*5.3 is mean and for HGD, 4.6 for LGD.
